# Linear Dynamic Sparse Modelling for functional MR imaging

**DOI:** 10.1007/s40708-014-0002-y

**Published:** 2014-09-06

**Authors:** Shulin Yan, Lei Nie, Chao Wu, Yike Guo

**Affiliations:** 1 Data Science Institute, Imperial College London, London, UK; 2 Institute of Computing Technology, Chinese Academy of Sciences, Beijing, China

**Keywords:** Linear Dynamic Sparse Modelling, Kalman filter, Sparse Bayesian Learning, Mutual information

## Abstract

The reconstruction quality of a functional MRI sequence is determined by reconstruction algorithms as well as the information obtained from measurements. In this paper, we propose a Linear Dynamic Sparse Modelling method which is composed of measurement design and reconstruction processes to improve the image quality from both aspects. This method models an fMRI sequence as a linear dynamic sparse model which is based on a key assumption that variations of functional MR images are sparse over time in the wavelet domain. The Hierarchical Bayesian Kalman filter which follows the model is employed to implement the reconstruction process. To accomplish the measurement design process, we propose an Informative Measurement Design (IMD) method. The IMD method addresses the measurement design problem of selecting *k* feasible measurements such that the mutual information between the unknown image and measurements is maximised, where *k* is a given budget and the mutual information is extracted from the linear dynamic sparse model. The experimental results demonstrated that our proposed method succeeded in boosting the quality of functional MR images.

## Introduction

Functional MR imaging (fMRI) technique has been widely used for measuring brain activity. By using controlled stimulus, it collects a sequence of brain MR images in order to localise brain activity which relies on neuron activity across the brain or in a specific region [1]. After being stimulated, the neurons remain active for only 4–6 s, so the time available for measuring neuron signals is physically constrained. In addition, the time for each measurement of a frequency by MRI is usually fixed [2], so the number of measurements that can be made is limited. For this reason, an urgent problem of fMRI is how to optimise the image quality using a limited number of measurements; two fundamental problems need to be addressed: How to boost the reconstruction by improving the reconstruction algorithm, and how to gather more information via a well-designed measurement strategy.

With a limited number of measurements, the image quality of MRI has been greatly improved using an emerging technique known as compressive sensing (CS). CS can reconstruct a signal accurately using underdetermined measurements as long as the signal can be sparsely represented in a specific domain [3]. Most of the existing CS methods guarantee an exact or approximate reconstruction if the measurement matrix which is determined by the measurement strategy is well-conditioned (e.g. satisfies RIP condition [3]). However, Sparse Bayesian Learning (SBL) [4], an advanced Bayesian CS method, does not have such strict requirement on the measurement matrix. Three different ways have been proposed to solve the MR imaging problem by utilising the CS techniques. The most direct way [2] is to apply CS to each MR image separately, while the quality of images reconstructed in this way is usually low. An alternative [5, 6] is to treat the entire sequence of MR images as a single spatiotemporal signal and perform CS to reconstruct it. The image quality obtained in this way is better, but a real-time reconstruction is impossible. The most recent and advanced way [7–9] is to employ dynamic tracking techniques to causally and sequentially reconstruct the images in an fMRI sequence, and therefore real-time reconstruction is realised. It greatly utilises the correlations of sparse patterns between two time-adjacent MR images so as to improve the reconstructed image quality.

In addition to the reconstruction algorithm, the image quality is also determined by measurement strategies; if the measurements carry more useful information about the signal, a higher quality image should be reconstructed. The most common measurement design scheme for the CS MR imaging technique is variable density random undersampling [2]. It chooses measurements according to a prior distribution which is calculated using distinct characteristics of signals in high and low frequency domains. In addition, historical MR images have been also used as prior information to design measurement trajectories [10, 11]. Moreover, Seeger et al. [12] designed an iterative Bayesian method to select measurements. In each iteration step, the posterior distribution of a MR image was updated using previous measurements. The new measurement was selected to minimise the uncertainty of the posterior distribution.

Most of the above methods are investigated for improving MR image quality. However, further improvement can be made in functional MRI. This is because it is a specialised application of MRI techniques which has some special properties (e.g. correlation exists between two time-adjacent functional MR images). In this paper, our work relies on a key assumption that variations of functional MR images are sparse over time in the wavelet domain. Based on this assumption, we first introduce the concept of linear dynamic sparse model; it is to model an fMRI sequence as a linear dynamical system with an identity transition matrix, and the image variations presented by the system noise are assumed to be sparse. Then, a linear dynamic sparse modelling (LDSM) method is proposed to solve the fMRI sequence reconstruction problem. Our LDSM method consists of two processes: image reconstruction and measurement design; both algorithms are investigated to fit the linear dynamic model.

Hierarchical Bayesian Kalman filter (HB-Kalman) [13] which is implemented by integrating CS with standard Kalman filter is an advanced dynamic sparse signal tracking algorithm. It sequentially reconstructs a signal sequence following our linear dynamic model, we therefore use it to implement the image reconstruction process of our LDSM method. The HB-Kalman algorithm employs the state-of-art CS method, SBL [4], to estimate the sparse variations between two adjacent images; the classic Kalman filter update step is processed for image reconstruction. The HB-Kalman algorithm can not only improve the point estimation of functional MR images, but also provide a full posterior density function (pdf) which yeilds “error bars” on the estimated image. These “error bars” can indicate the measure of confidence of the reconstructed image. Our measurement design method, Informative Measurement Design (IMD), makes use of the posterior distribution of the reconstructed image. It is the first measurement design method in fMRI that utilises the correlations of fMRI images in a sequence. It calculates the prior distribution of the present image using the posterior distribution of the previous adjacent image as well as the prior distribution of image variations. After obtaining the prior distribution of an unknown image, the measurement design problem turns to select *k* feasible measurements, where *k* is a given budget. The measurements are selected to maximise the mutual information [14] between the unknown image and measurements. As this problem is intractable, a novel approximation method is employed to solve it. Comparing with the previous fMRI methods, our approach makes better use of signal information so that the qualities of reconstructed images can be highly increased.

The remaining paper is organised as follows: In Sect. 2, we first formulate the fMRI sequence reconstruction problem using a linear dynamic sparse model. We then illustrate our LDSM method and explain both the reconstruction and measurement design algorithms in Sect. 3. Next, the experiment results of applying our method to an fMRI sequence are detailed in Sect. 4. Finally, Sect. 5 presents discussions of our work.

## Problem formulation

### Sparsity of variations

**Fig. 1:**
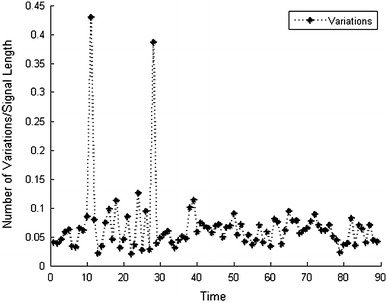
Example of sparse variations

The key assumption of our work is that the variations of functional MR images are sparse over time in the wavelet domain. We demonstrate it for a fMRI sequence [15] in Fig. 1. In order to reduce the impact of measurement noise, the variations are filtered by a threshold which is determined by one-tenth the maximum variation in a given time interval. The sparsity level is determined by $|N_{c}\backslash N_{t}|$, where *N*_*t*_ refers to the number of two-level Daubechies-4 2D discrete wavelet transform (DWT) of the functional MR image at time *t*, and $N_{c}=|N_t\backslash N_{t-1}|$ refers to the number of DWT coefficient changes with respect to the previous frame. In most cases, the number of variations is less than 10 % of the signal size. Note that the two outliers $(|N_{c}\backslash N_{t}|>40\,\%)$ result from the high degree of similarity between the two time-adjacent images. When the two images are nearly the same, the maximum variation is so small that the noise impact is increased.

### Linear Dynamic Sparse Model

Linear dynamic model [16] is a state-space model that describes the probabilistic dependence of a latent variable and its corresponding measurements. It is characterised by a pair of equations: system equation and measurement equation. Our proposed linear dynamic sparse model is a special case of linear dynamic model, that is, the system equation is modified to meet the sparsity constraint. The details of our model are explained below.

#### System equation

Based on the assumption that the variations of functional MR images are sparse, an fMRI sequence is modelled as a linear equation with an identity transition matrix:

1 $$\begin{aligned} x_t = x_{t-1} + q_t, \end{aligned}$$

where random variable*x*_*t*_ denotes the DWT coefficients of a functional MR image at time *t*. For simplicity, we call *x*_*t*_ image in the rest of this paper. Random variable *q*_*t*_ denotes its sparse variations with respect to the previous image *x*_*t*−1_. To meet the sparsity constraint, a hierarchical sparseness prior is placed on *q*_*t*_. Each element *q*_*ti*_ of the variation *q*_*t*_ is randomly sampled from a zero-mean Gaussian distribution $N(q_{ti}|0,\alpha _i^{-1}),$ the variance *α*_*t*_ of which is randomly sampled from a Gamma $\varGamma (\alpha _i|a,b).$ That is,

2 $$p(q_t|a,b) = \prod_{i=1}^{N_t}{\int_0^\infty{\mathcal{N}(q_{ti}|0,\alpha_i^{-1})\Gamma(\alpha_i|a,b)\mathrm{d}\alpha_i}}. $$

After marginalising the hyperparameter, the prior of *q*_*t*_ corresponds to a product of independent student’s *t* distribution. Tipping et al. [4] demonstrate a strong sparse property of this hierarchical distribution.

#### Measurement equation

The fMRI technique measures a subset of discrete Fourier transform (DFT) coefficients of MR images. At each time *t*, the measurement process can be modelled as:

3 $$\begin{aligned} y_t = \varPhi _t x_t + n_t, \end{aligned}$$

where random variable *y*_*t*_ which is called measurements in this application is a subset of DFT coefficients determined by the measurement matrix $\varPhi _t,$ and random variable *n*_*t*_ refers to the measurement noise. The measurement matrix $\varPhi _t$ is formed by a subset of *k* vectors selected from the projection matrix $\varPhi$, which in our case is constructed by the DFT matrix and the inverse DWT matrix. The budget *k* is a given positive integer. It determines the number of frequencies to be measured.

## Methods

**Fig. 2:**
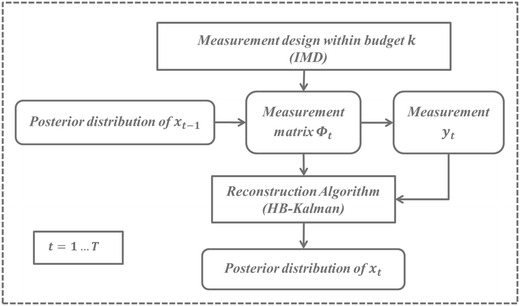
Framework of fMRI sequence reconstruction

Our proposed LDSM method aims to design measurement strategy as well as reconstruct image sequence following the linear dynamic sparse model. Figure 2 illustrates the framework of our method. For each time instance, the measurement design method is first performed to select a subset of *k* vectors from the projection matrix $\varPhi$ using the posterior distribution of the previous adjacent image *x*_*t*−1_, where the selected vectors are used to form the measurement matrix $\varPhi _t.$ When the measurement *y*_*t*_ is obtained by following the determined strategy, the posterior distribution of the present image *x*_*t*_ can be calculated using the reconstruction algorithm, and it can be used in the next measurement design process. The framework is processed iteratively until the whole fMRI sequence is reconstructed.

HB-Kalman (see [13] for more details) which is derived from the principles behind the Kalman filter and SBL is employed to implement the image reconstruction process. It works on the linear dynamic sparse model and meets the sparsity constraint. Benefiting from the hierarchical Bayesian model, the posterior distributions of reconstructed images are provided which satisfy the requirement of the measurement design process.

The reconstruction quality of a functional MR image is limited by the information obtained from measurements. According to [14], information acquired from measurements can be quantified by the mutual information between the unknown image and measurements. The mutual information quantifies the extent to which uncertainty of the unknown signal is reduced when measurements are given. Furthermore, measurements are determined by a measurement matrix according to the measurement equation (Eq. 3). Given the budget *k* (the number of DFT coefficients to be measured), the measurement design problem is to select a subset of *k* vectors from the projection matrix $\varPhi$ so as to maximise the mutual information between the unknown image and measurements, where the mutual information is defined as follows:

4 $$\begin{aligned} I(x_t;y_t) = h(y_t) - h(y_t | x_t). \end{aligned}$$

Because the conditional entropy $h(y_t |x_t)$ is merely the entropy of noise *n*_*t*_, which is an invariance to the measurement matrix $\varPhi _t,$ we can maximise the entropy *h*(*y*_*t*_) of the measurements *y*_*t*_ instead. Using the system equation (Eq. 1) and the measurement equation (Eq. 3), we obtain:

5 $$\begin{aligned} y_t = \varPhi _t(x_{t-1}+q_t) + n_t. \end{aligned}$$

Because *n*_*t*_ is invariant to $\varPhi _t,$ maximising*h*(*y*_*t*_) is equivalent to maximising $h(\varPhi _t(x_{t-1}+q_t)).$ The measurement design problem then addresses the solution of the following optimisation problem:

6 $$\begin{aligned} &\varPhi _t = \hbox {arg max}_{\varPhi _t}h(\varPhi _t(x_{t-1}+q_t)) \\ &\text{s.t.} \, \varPhi _t \, \text{is formed by} \;k \;\text{row vectors of}\; \varPhi . \end{aligned}$$

The posterior distribution of *x*_*t*−1_ which is provided by the HB-Kalman reconstruction algorithms is a multivariate Gaussian distribution with mean $x_{t-1|t-1}$ and covariance $\varSigma _{t-1|t-1}.$ As explained in the system equation (Sect. 2.2.1), we place a student’s *t* sparse prior on each element of *q*_*t*_. To make the prior non-informative, we set the hyperparameters *a* and *b* close to zero. Given the posterior distribution of *x*_*t*−1_ and the prior distribution of *q*_*t*_, the distribution of *y*_*t*_ can be determined. However, the calculation of close form of the sum of a norm random variable and a student’s *t* random variable is analytically intractable. Seeger et al. [17] suggested that a student’s *t* distribution can be approximated in terms of a Gaussian distribution, we therefore use a zero-mean multivariate Gaussian distribution to approximate the sparse prior of *q*_*t*_, where $q_t\sim \prod _1^N{\mathcal {N}(0,c)}.$ The constant value *c* is determined by the level of variations *q*_*t*_. The higher the level, the larger the value of *c* should be.

As *y*_*t*_ is an affine transformation of $$(x_{t-1}+q_t)\sim \mathcal {N}(x_{t-1|t-1},\varSigma _{t-1|t-1}+{\text{diag}}(c)),$$$$\varPhi _t(x_{t-1}+q_t)$$ has a multivariate normal distribution with mean $$\varPhi _t x_{t-1|t-1}$$ and covariance $$\varPhi _t(\varSigma _{t-1|t-1}+{\text{diag}}(c))\varPhi _t^T.$$ The entropy $$h(\varPhi _t(x_{t-1}+q_t))$$ therefore satisfies

7 $$\begin{aligned} &\varPhi _t = \hbox {arg max}_{\varPhi _t} || \varPhi _t(\varSigma _{t-1|t-1} + {\text{diag}}(c))\varPhi _t^T|| \\ &{\text{s.t.}} \, \varPhi _t \, {\text{is formed by}} \, k \, {\text{row vectors of}} \; \varPhi . \end{aligned}$$

Solving the above optimisation problem usually has high computational complexity. For this reason, an approximation approach [18] is employed. Because the objective function is submodular, this method does not only reduce the computational complexity but also provide performance guarantee.

In each iteration *l*, this algorithm is to select one row $\varPhi _{S^*}$ from the unselected set $\varPhi _{S^l}$. The selected row is the solution of this following optimization problem:

8 $$\begin{aligned} s^* \leftarrow \hbox{arg max}_{j\in S^l}\phi _jU^{-1}_l\phi _j^T \\ {\mathrm{with}} \,\, U_l = \sigma ^{-1}\sum _{i\in M^l}\phi _i^T\phi _i+\varSigma _{t|t-1}^{-1}, \end{aligned} $$

where *S*^*l*^ and *M*^*l*^ denote the unselected and selected projection vectors before iteration *l*, respectively, and where $\varSigma _{t|t-1} = \varSigma _{t-1|t-1}+{\text{diag}}(c).$

Our proposed method, IMD, not only uses the posterior distribution of the previous signal to model the uncertainty of the current unknown signal, but also involves a sparse prior of the variation signal to further modify the uncertainties. The measurement matrix is constructed by *k* numbers of projection vectors selected from the projection domain, and the determined measurements can improve the reconstruction accuracy.

## Experimental results

We performed experiments on a fMRI sequence used by Lu et al. [15], which was generated by a real rest brain sequence with additional synthetic BOLD contrast. The rest brain sequence $(\hbox {TR/TE}=2,500/24.3\,\text{ms},\, 90^{\circ }$ flip angle, 3 mm slick thickness, 22 cm FOV, 64 × 64 matrix, 90 volumes) was acquired by a 3T whole-body scanner and a gradient-echo echo-planar imaging (EPI) acquisition sequence. The BOLD contrast signal convolved with a bi-Gamma hemodynamic response (HDR) was created to represent a 30-s on/off stimulus, and it was added to the pixels at an average contrast-to-noise ratio (CNR) of 4.

Two experiments were conducted to reconstruct the first 15 volumes of the image sequence with *k* = 0.3*N* measurements for*t* > 1. The reconstruction accuracy is evaluated according to the root squared error (RSE), defined as $e(t)=||x_t-\hat{x}_t||_2/||x_t ||_2$. In the first experiment, with *k* randomly selected measurements, we compared the reconstruction accuracies obtained using the HB-Kalman algorithm and the SBL algorithm [4], and demonstrated that HB-Kalman performed better. Then, in the second experiment, we used the HB-Kalman to reconstruct the fMRI sequence. We applied our proposed measurement design method to select *k* measurements, and compared it against the random selection technique.

### HB-Kalman versus SBL

**Fig. 3:**
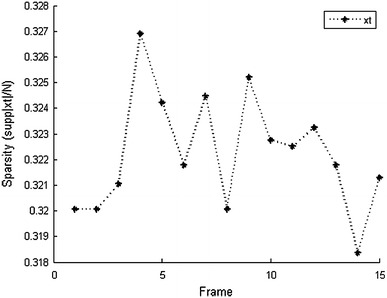
Sparsity of image *x*_*t*_. $|{\text{supp}}(x_t)|$ refers to the 95 % energy support of DWT coefficients of image at time *t*

**Fig. 4:**
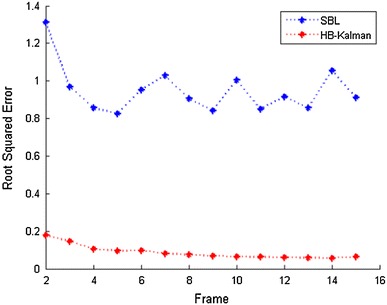
Reconstruction errors (HB-Kalman vs. SBL)

We compare the performances of HB-Kalman and SBL. SBL reconstructs the image sequence by performing a simple SBL process on each MR image. SBL is comparable to HB-Kalman, as it is a CS method and satisfies the requirement of our method that it can estimate a posterior distribution of the unknown image. Both methods carry out the reconstruction process with a limited number of random samples. From Fig. 4, we can clearly see that the SBL algorithm generates nearly random guesses. This is because the wavelet transform coefficients are not very sparse (as shown in Fig. 3, $|{\text{supp}}(x_t)|\approx 31\%N$), so the underdetermined observations $(k=30\,\%N)$ cannot provide enough information of the unknown signal to even produce a rough reconstruction result. By contrast, HB-Kalman has remarkable reconstruction performance. It uses the knowledge of the preceding image as a prior to predict the present functional MR image, and the observations are used to modify the prediction. Hence, even when the samples are under-determined, the information is large enough to provide an approximate or exact reconstruction result.

### IMD versus random sampling

The above result demonstrated that the HB-Kalman reconstruction algorithm performed better on the fMRI application. We used HB-Kalman to implement the reconstruction process, and focused on comparing the reconstruction performances by utilising random sampling and the IMD method. The constant value in Eq. 7 is empirically set to $c=1e^2$.

**Fig. 5:**
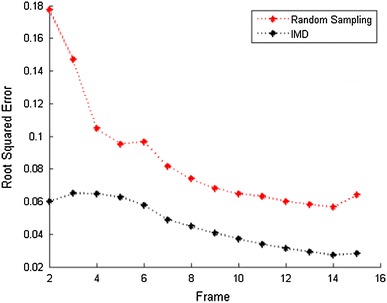
Reconstruction errors (IMD vs. random sampling)

The results shown in Fig. 5 demonstrate a significant improvement in the reconstruction accuracy from random sampling to the IMD. The reconstruction error of the IMD method is in average 46.28 % less than when using random sampling (45.2 vs. 86.5 %).

It is worthwhile to point out that both methods have a decreasing trend of reconstruction errors in the number of frames. This is because the brain images are very similar to each other. As the number of reconstructed frames (the total number of samples) increases, the uncertainty of the unknown frame is reduced.

**Fig. 6:**
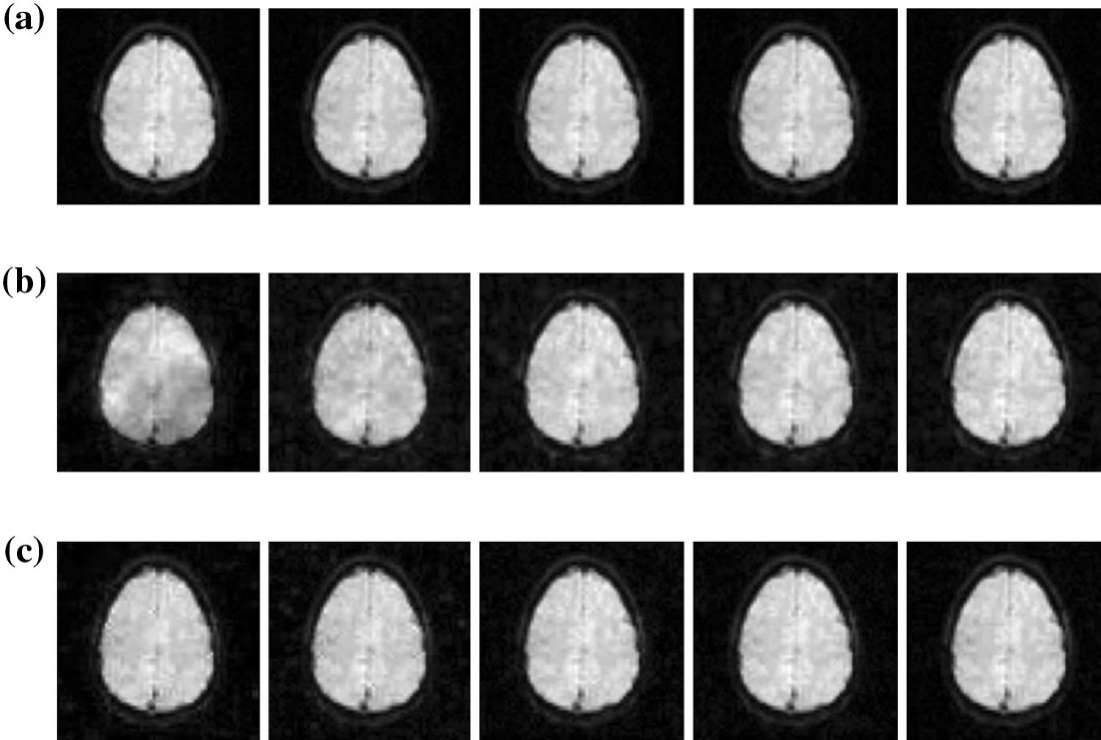
Reconstructions of functional MR images (2nd,  5th,  8th,  11th,  14th frames). **a** original sequence, **b** random sampling, **c** proposed method

Furthermore, Fig. 6 shows the visually reconstructed results generated by the two methods. The random sampling results in more blurry and noisy functional MR images. Meanwhile, the IMD method is able to provide more detailed functional MR images, which is very important in fMRI techniques (e.g. activity pattern detection).

## Discussion and conclusion

In this paper, we propose a LDSM method for solving functional MRI sequence reconstruction problem. Based on a key assumption that the variations of functional MR images are sparse over time in the wavelet domain, our method models an fMRI sequence as a linear dynamic sparse model. By using the linear sparse model, the prior information of the unknown fMRI image can be extracted from the previous fMRI image and the sparse variations. The prior information, expressing certainty and uncertainty of an unknown image, can be employed to boost the reconstructed image quality. Firstly, the uncertainty of the image can be used to guide the measurement so that more useful information can be obtained. Secondly, even when the number of measurements is under-determined, a high quality image can still be generated by involving its prior information in the reconstruction process. For this reason, the reconstruction and measurement design algorithms that adopt the linear dynamic sparse model are preferred.

In fMRI, measurements are achieved by following predefined acquisition trajectories. The early trajectory used in MR imaging is Cartesian. It generates images with few artefacts, but its long acquisition time is against the speed requirement of fMRI. Then, a rapid acquisition trajectory, echo planar imaging (EPI), was started to be concerned in 1977 [19]. Comparing with Cartesian, the acquisition speed is highly increased. However, it results in longer readout duration so that artefacts are introduced. More recently, spiral trajectory has regained interest with applications of fMRI [20]. It makes fast and efficient use of gradient hardware, and introduces fewer artefacts by reducing the readout duration. The conventional reconstruction methods for spiral trajectory [21, 22] require interpolation of the raw data and consume long computational time, e.g. several hours and sometimes even days. The most recent reconstruction methods, based on CS, are promising to overcome the computational limitation. They work with underdetermined measurements and require the measurements to be incoherent; random sampling is usually used, as it can provide low incoherent measurements. To satisfy the requirement of reconstruction methods, the development of advanced trajectories has been continued to be driven by neurosciences, and more powerful and higher field strength systems have become available [23, 24]. Our proposed IMD method aims to find a small number of measurements that are maximally informative about the signal. Comparing with random sampling, our method can generate more informative measurements, with which higher quality images are achieved. Our method has the potential to be developed by modifying the spiral trajectory. The spiral trajectory enables sparse acquisition methods, and the candidate measurements provided by it are individual voxels rather than parallel lines of *K*-space that are provided by Cartesian trajectories. In addition, multi-interleave-perturbed spiral trajectory [23] can cover approximately the full *K*-space which is desired by our method.

The IMD method is an extension of the Bayesian method of Seeger et al. [12] that utilises correlations of adjacent images in an fMRI sequence. This is the first study to explore the benefits of this for designing measurements. Two approximation techniques are used in this study to resolve the intractability of the measurement design problem. One is to use a zero-mean multivariate Gaussian distribution to approximate the student’s distribution, which makes the calculation of the prior distribution of a MR image tractable. The other is to use a greedy algorithm to reduce the computational complexity of the optimization problem. The experiment results demonstrate that our proposed method can improve the quality of reconstructed functional MR images. However, the theoretical bounds of the approximation techniques are still unknown. Also, a learning algorithm, that can enable dynamic modification of the hyperparameters of variations using the information from reconstructed images, needs to be explored in the future.
